# Electron energy analysis by phase-space shaping with THz field cycles

**DOI:** 10.1063/1.5045167

**Published:** 2018-08-29

**Authors:** Dominik Ehberger, Catherine Kealhofer, Peter Baum

**Affiliations:** 1 Ludwig-Maximilians-Universität München, Am Coulombwall 1, 85748 Garching, Germany; 2 Max-Planck-Institute of Quantum Optics, Hans-Kopfermann-Str. 1, 85748 Garching, Germany; 3 Department of Physics, Williams College, Williamstown, Massachusetts 01267, USA; 4 Fachbereich Physik, Universität Konstanz, Universitätsstr. 10, 78467 Konstanz, Germany

## Abstract

Time-resolved electron energy analysis and loss spectroscopy can reveal a wealth of information about material properties and dynamical light-matter interactions. Here, we report an all-optical concept for measuring energy spectra of femtosecond electron pulses with sub-eV resolution. Laser-generated terahertz radiation is used to measure arrival time differences within electron pulses with few-femtosecond precision. Controlled dispersion and subsequent compression of the electron pulses provide almost any desired compromise of energy resolution, signal strength, and time resolution. A proof-of-concept experiment on aluminum reveals an energy resolution of <3.5 eV (rms) at 70-keV after a drift distance of only 0.5 m. Simulations of a two-stage scheme reveal that pre-stretched pulses can be used to achieve <10 meV resolution, independent of the source's initial energy spread and limited only by the achievable THz field strength and measuring time.

## INTRODUCTION

Ultrashort electron pulses of femtosecond and attosecond duration allow direct visualization of the fastest atomic and electronic processes in matter with simultaneous resolution in space and time.^1,2^ Diffraction patterns of crystalline materials or real-space images of complex morphologies obtained in a pump-probe way have already elucidated numerous ultrafast phenomena in gaseous, solid, and liquid environments,^3–8^ and researchers are continuing to explore even more fundamental processes^9^ or more complex materials.^10^

Besides the tremendous spatial and temporal resolution offered by the latest technology advances,^11–16^ there is also much useful information hidden in the energy distribution of the probing electrons. Electron energy loss spectroscopy (EELS; see Ref. 17 for a review) is therefore a widespread method to obtain fundamental chemical and structural information from complex materials. For example, plasmonic^18^ or vibrational excitations^19^ can be probed with spectral and spatial resolution of ∼10 meV and <1  nm at the same time.^19^ Recently, the capabilities of EELS have also been extended to the femtosecond domain.^20^ Even core-loss spectra can be recorded with nanosecond temporal resolution.^21^

An energy analyzer for electrons at energies of tens to hundreds of keV is central to these types of experiments. The desired resolution is <1 eV for studying laser-electron interactions^22–25^ and ideally meV for studying phonon excitations^19,26^ or details of plasmonic effects. Such resolution is typically achieved by rather bulky and expensive analyzers based on deflection magnets and chicanes. Also, those magnetostatic analyzers put stringent restrictions on the beam quality that are difficult to meet with laser-generated ultrashort electron pulses such as required for time-resolved investigations.

Time-of-flight (ToF) energy analyzers, on the other hand, measure the relative arrival times of particles in a beam after some drift distance and are, in principle, not limited by finite emittance. However, the nanosecond-scale response of state-of-the-art electronics has restricted the applicability of the ToF concept to rather low electron energies in the sub-keV regime.^27^ Recently, 0.5-eV resolution of 30-keV pulses has been achieved by deceleration of the beam to a drift energy of only a few eV.^28^

The close relation between time and energy in femtosecond electron pulses offers another, dynamical approach for energy analysis.^29^ The idea is to measure arrival time differences with femtosecond instead of nanosecond resolution. Additionally, smart phase-space shaping prior to temporal detection allows for substantial improvement of the energy resolution at cost of time resolution.^30^ A first demonstration of such type of ultrafast time-of-flight analysis was made with a series of microwave cavities^29,30^ that are synchronized to each other.

Here, we apply all-optically generated THz pulses for time-of-flight electron energy analysis. This approach avoids the need for temperature stabilization^31^ or active synchronization^32,33^ to a femtosecond laser for pump-probe investigations. Throughout the experiment, the mean electron energy is left unaltered and thus no deceleration into a drift stage is required. The necessity of a magnetically shielded environment is largely reduced, because magnetic beam deflection scales inversely with the electron velocity. In addition, THz pulses are naturally synchronized to femtosecond lasers,^12^ facilitating high-resolution pump-probe investigations.

## CONCEPT FOR THz-BASED TIME-OF-FLIGHT DETECTION

**FIG. 1.**
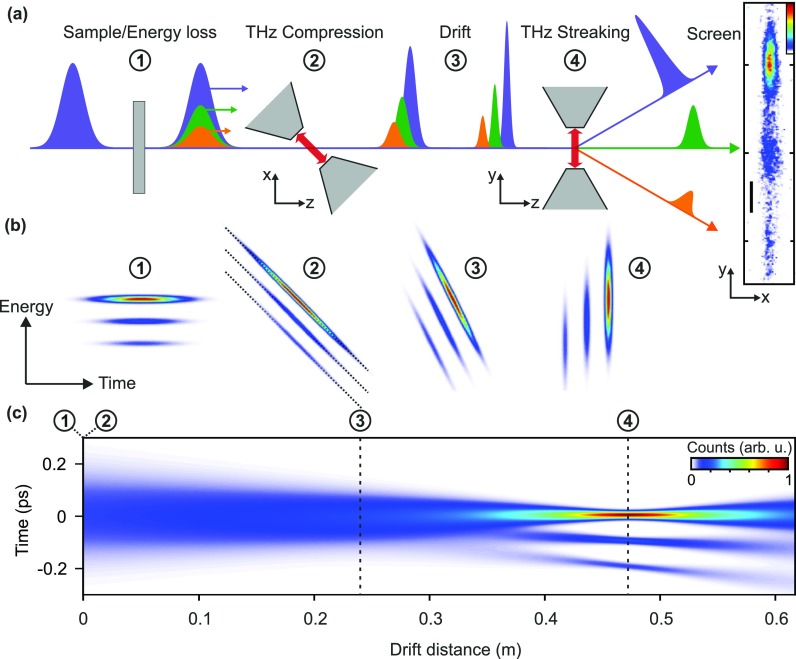
Setup and concept. (a) An electron pulse (purple) is modulated in energy via interaction with a sample (①). The pulse passes through a bow-tie resonator irradiated with a THz pulse and obtains a time dependent, longitudinal momentum modulation (②) that leads to compression upon further propagation (③). Electrons with different forward velocities (green, orange) are separated in time while being compressed. Temporal characterization in the temporal focus via streaking with a THz-illuminated bow-tie resonator (④) allows discriminating electrons of different initial energy on a screen (right panel; scale bar corresponds to 0.5 mm; linear color scale from 0 to 1 in arbitrary units). (b) Energy-time phase-space picture of this time-of-flight electron energy analysis with temporal lenses. Dotted lines indicate the compression slope. (c) Simulated evolution of the temporal profile of a 250-fs electron pulse with hypothetical energy losses of 5 eV and 10 eV. The initial energy spread of the source is set to 1 eV FWHM and we assume linear phase space transformations and no electron-electron interactions. The temporal focus is 46.8 cm behind the compression element, as in the experiment.

Figure 1(a) depicts the principle of operation of our THz-ToF detector and its essential components. An initial electron pulse (purple) from a laser-triggered photocathode interacts with a sample (grey) and thereby modulates the spectrum of the electron pulses in a material-specific way (green and orange pulses). Subsequently, the energy-modulated parts of the pulse travel at different mean velocities. The resulting arrival time differences are detected after a certain drift distance with femtosecond accuracy via streaking with THz-pulses.^12^ As compared to conventional, electronic time-of-flight detectors, we have here 10^3^–10^4^ times better resolution. This enhancement allows us to avoid any deceleration and few-eV drift stage.

Compression of the electron pulse in time can further enhance the resolution, because shorter electron pulses can better be detected in time. For compression, the electrons pass through a bow-tie shaped metal resonator (grey) again driven by THz field cycles. They obtain a longitudinal momentum kick, which varies in strength as a function of arrival time. By setting the timing such that earlier electrons in the pulse are decelerated and later ones accelerated, the resonator acts as a focusing lens in time.^12^ It creates a correlation between a particle's position within the pulse (proportional to time) and its forward momentum (approximately proportional to energy). There is an analogy to optical lenses which establish a correlation between transverse beam position and angle with the optical axis.^34^ Accordingly, a temporal focus—defined as the point in space of minimum pulse duration—is obtained at some drift distance from the compression element. If the electron spectrum is modulated, each part is focused approximately to the same temporal focus, but arriving at a different time. This temporally mapped spectrum is detected by the streaking of the beam with a second bow-tie resonator.^12^ In the end, on a screen behind the streaking element, we detect a distribution of particle positions that reveals the energy spectrum.

Figure 1(b) shows a phase-space representation of the concept. We start with an electron pulse with a zero loss peak and two different energy loss peaks (①). Next, there is the phase space transformation by the temporal lens for compression (②). Earlier electrons are slower than later ones. Next, the phase space distribution shears under propagation (③) until it is rotated by 90° with respect to its initial state. This position is the temporal focus (④). Here, measurements of distributions of electron arrival times via streaking become measurements of the initial energy distribution. Figure 1(c) depicts the evolution of the temporal profile on the pulses' way to the temporal focus. At this position, the energy loss peaks are well separated from the zero loss peak.

Conversion from time to energy is approximated by linearization of the kinematic equations for relativistic electrons. The approximation is valid if the energy loss δE (typically <100 eV) is much smaller than the mean kinetic energy $E_{0}$ of the beam (typically 30–300 keV). Using the Lorentz factor $\gamma ={\left(1-\beta^{2}\right)}^{-1/2}=1+E/(m_{e}c^{2})$, the electron velocity $v=\beta c=c\sqrt{1-\gamma^{-2}}$, the speed of light c, and the electron mass $m_{e}$, we obtain

$$\frac{dv}{dE}=\frac{\partial v}{\partial \gamma }\frac{d\gamma }{dE}=\frac{c}{\beta \gamma^{3}}\frac{1}{m_{e}c^{2}}\;$$ (1)

and

$$\frac{dt}{dv}=-\frac{d}{v^{2}}.$$ (2)

With knowledge of $E_{0}$ and the distance d between compression and streaking, we can convert arrival time differences δt to differences in energy via

$$\delta E\approx \frac{\beta_{0}^{3}\gamma_{0}^{3}c^{3}m_{e}}{d}\delta t,$$ (3)

where $\gamma_{0}=\gamma (E_{0})$ and $\beta_{0}=\beta (\gamma_{0})$.

Equation (3) also holds if there is a finite propagation distance between the sample and the compression element. Although the energy loss pattern ① further disperses in time, the compressor offsets this effect. If electrons at a given energy arrive earlier or later, they obtain a correspondingly greater or lower longitudinal momentum kick and always arrive at the temporal focus at the same time.^35^ Basically, the distribution ② just shifts along the dotted lines. The analogy to the optical lens is apparent: it does not matter at which transverse position (time) the lens is hit, only the angle (energy) determines the transverse position of (arrival time at) the focus (temporal focus).

## EXPERIMENTAL SETUP

In the experiment, we use frequency-doubled laser pulses from a 1-ps, Yb:YAG thin-disk laser with a central wavelength of 1030 nm [Ref. 36] at 50 kHz to trigger electron emission from a back illuminated flat gold cathode that is held at −70  keV with respect to a grounded anode that is 25 mm apart.^37^ The same laser source is used to generate single-cycle THz pulses at 0.3 THz central frequency^36,38^ for compression and streaking with bow-tie resonators.^12^ Both resonators are designed to resonantly match the central frequency of the driving THz pulses. In the center, the resonators have a clear aperture of 80 *μ*m. Two magnetic solenoid lenses, one before and one after the compression element are used to steer the electron beam through the resonators without clipping. Space charge effects are largely circumvented by using less than 10 electrons per pulse. The electron pulses first pass through a ∼70 nm thick freestanding Al foil where they lose energy by inelastic plasmon scattering; the total transmission through the foil is ∼30%. After propagation for 2 mm, the pulses arrive at the first bow-tie resonator which serves as compression element. It is rotated by ∼45° around the *y* axis [see Fig. 1(a)]. The electron and the THz beams cross approximately at right angle. This configuration provides a non-zero component of the electric field along the electrons' trajectory.^12^ The THz peak field strength impinging onto the first resonator is $∼5\times 10^{5}$ V/m in order to let the temporal focus coincide with the position of the second resonator used for streaking at a drift distance of d=(46.8±0.5) cm. The THz peak field strength driving the second resonator is $∼1\times 10^{6}\;$ V/m. The streaking resonator is excited collinearly with the electron beam. This configuration leads to a sideways deflection of the electron beam which is detected by a camera equipped with a scintillator screen.^39^ In order to enhance the streaking resolution, we placed an aperture of 50 *μ*m diameter between compression and streaking stage.

**FIG. 2.**
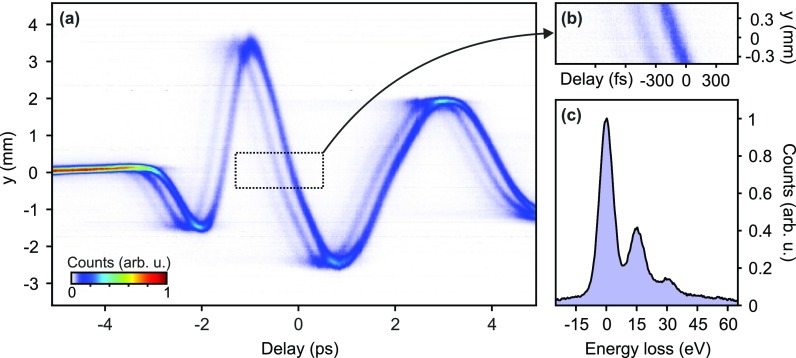
Experimental results. (a) Deflectogram of the compressed electron pulses after passage through a ∼70-nm thick aluminum sample. The two shadow traces indicate the energy loss due to inelastic plasmon scattering. (b) Details of the deflectogram. The loss traces are each delayed by ∼300 fs. (c) Measured electron energy loss spectrum of the Al foil. There are peaks at multiples of 15 eV, corresponding to the bulk plasmon energy.

Streaking profiles are obtained by integration of the screen images along the unstreaked dimension [*x*-axis, see Fig. 1(a)]. Alternatively, this screen direction could also be used for position-dependent EELS imaging. Figure 2(a) shows a series of the measured streaking profiles as a function of the delay of the streaking THz field with respect to the electrons. We call such a representation a deflectogram.^12^ The center of mass of the deflected electron pulse follows the streaking field inside the resonator. Around the zero crossing [dotted rectangle in Fig. 2(a)], we measure a temporal resolution of 12 fs (rms). Via Eq. (3), this time resolution corresponds to an energy resolution of 0.6 eV.

## RESULTS

In the deflectogram of Fig. 2(a), we see a prominent zero loss deflection trace in addition to several weaker traces that are each delayed by roughly 300 fs. Figure 2(b) shows a close-up of the region around the zero-crossing in Fig. 2(a) that is marked with a dotted rectangle. We clearly see three distinct features that are delayed in time. Averaging along these traces and application of the time-energy conversion according to Eq. (3) yields the energy spectrum depicted in Fig. 2(c). We find two energy loss peaks at (14.9±0.2) eV and (29.9±0.3) eV. A third peak may be hidden in the background.

Aluminum's bulk plasmon has an energy of 15.0 eV.^17^ The two observed EELS peaks are therefore the first-order and second-order plasmon losses in Al. From the width of the measured zero-loss peak, as shown in Fig. 2(c), we infer an upper limit for the energy resolution of 3.5 eV (rms) of our THz-ToF, which corresponds to a relative accuracy of ${5\times 10}^{-5}$. Earlier measurements of electron pulse duration without the aluminum sample^12^ indicate an energy resolution in our setup of down to 1.7 eV (rms). The electron pulse duration at the Al sample is in the femtosecond regime and therefore the setup would, in principle, allow femtosecond pump-probe investigations.

## DISCUSSION

In the configuration presented above, the energy resolution is fundamentally limited by the bandwidth of the electron source [see Fig. 1(b)]. The typical bandwidths of laser-driven electron sources are 0.5–1 eV [Ref. 23] or larger, depending on the amount of space charge. For flat cathodes, the initial energy spread can be reduced by matching the cathode material's work function to the triggering photon energy,^37,40^ which, in principle, allows for a resolution of ∼0.1 eV. Electron beam monochromators like used in state-of-the-art electron microscopes^41^ would help to improve the resolution even further. Practically, the resolution in our scheme is limited by the ability to compress the electron pulses. While timing problems due to jitter are largely avoided by our all-optical approach,^12^ nonlinearities of the phase space transformations and spatiotemporal distortions arising from magnetic solenoid lenses^42^ are responsible for energy resolution imperfections. These temporal lens aberrations can be overcome by careful lens alignment,^43^ using shorter initial electron pulses, and by compression elements that allow for velocity matching.^23,44^

The finite cycle duration of the streaking THz field restricts the energy range that can be measured. For our experiment, the measurement window is ∼80 eV, considering the turning points of the streaking trace as the main limiting factor. This range is already enough for most experiments on plasmons or optical modulations, but if necessary it can be improved by decreasing the drift distance d and increasing the compression and streaking field strengths. The spectral features will be denser in time and thus a broader energy range will be covered by a single streaking half-cycle. Such an increase in detection range would, for example, allow femtosecond core-loss spectroscopy with keV-scale energy losses.

The potential compactness of our THz-ToF is one of its most prominent features. The length of the necessary beam line is solely determined by the available THz field strength. The drift distance is determined by the shortest achievable temporal focal length and the energy resolution scales with the THz power at the streaking element. Electron beam modulation has been demonstrated with THz peak fields exceeding 10^7^ V/m,^45^ about 20 times larger than in our experiment. With such sources, our THz-ToF could reach a length of merely centimeters.

## MULTI-STAGE THz-ToF FOR ENERGY RESOLUTION BEYOND THE CATHODE LIMIT

**FIG. 3.**
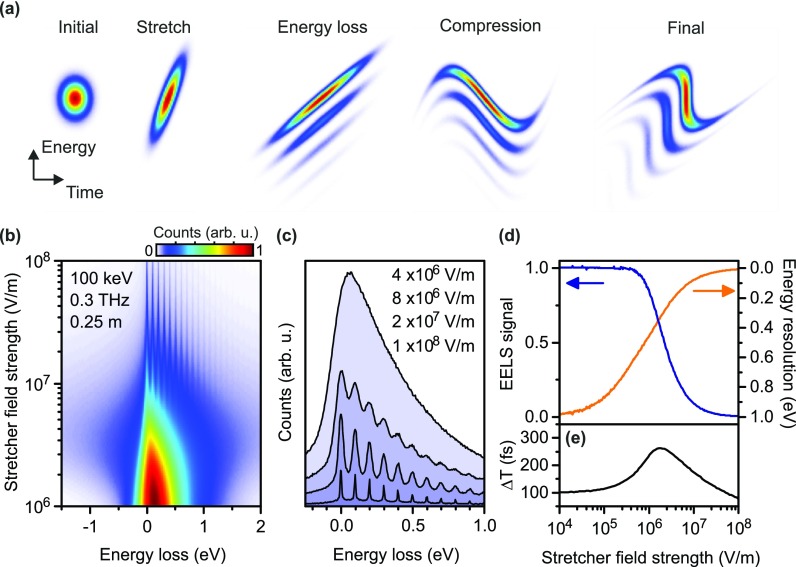
Simulation of a two-stage THz-ToF for increased energy resolution. (a) Sequence of phase space transformations. Here, nonlinearities of the THz fields at the compression stage are taken into account by assuming a single-cycle, sine-like velocity modulation. The compression therefore becomes, in part, nonlinear for strongly stretched pulses. (b) Electron energy loss spectra of hypothetical 100-meV features in an electron energy loss spectrum for different stretcher field strengths. The beam energy is 100 keV and modulation is made by single-cycle THz pulses with a central frequency of 0.3 THz. The source's initial energy spread and pulse duration are 1 eV and 100 fs (FWHM), respectively. The drift distance between stretcher and compressor is 0.25 m. (c) Resulting energy spectra for different stretcher field strengths as indicated by color. (d) Useful EELS signal (relative) and energy resolution (FWHM) as a function of the stretcher field strength. (e) Effective temporal resolution, ΔT, defined as the FWHM of the longitudinal electron distribution at the sample within the FWHM of the EELS peaks.

An energy resolution even better than the initial energy spread of the electron source can be achieved by adding one more THz-electron interaction stage, as originally proposed by Verhoeven *et al.* for microwave cavities.^30^ This THz stage acts a stretcher, that is, a negative temporal lens that accelerates the leading part of the pulse and decelerates its trailing part. A phase-space representation of this process is depicted in Fig. 3(a). Such a stretcher can, for example, be implemented with the well-established methods for compressors^12^ but with THz cycles of opposite slope. During subsequent propagation, the electron pulse spreads in time much faster than without the stretcher. If such a chirped electron pulse loses energy in an inelastic scattering process, the parts with the loss are also chirped in the same way. After compression and streaking, the energy resolution is enhanced by factor that depends on the amount of additional electron chirp. However, in this case, nonlinearities of the THz field cycles have to be considered.

In order to investigate this possibility, we simulated the evolution of an EELS dataset with a hypothetic 100-meV modulation (for example, phonon excitations) as a function of stretcher field strength. We assume single-cycle THz pulses at a central frequency of 0.3 THz. The electron pulses have an energy of 100 keV, 100 fs initial duration, and 1 eV energy spread (both FWHM). The distance between stretcher and sample is 0.25 m.

The simulation employs a Monte-Carlo algorithm, which calculates the final arrival times of $10^{7}$ classical particles after passing through the experiment. Each particle is defined by its velocity and longitudinal position and obtains a nonrelativistic velocity kick at the stretcher and compressor, which varies as a function of the arrival time. The strength of this sinusoidal modulation at the stretcher is varied and the strength of the compressor is chosen such that the temporal focus remains at 0.5 m. Energy loss at the sample is modeled by random, and uncorrelated velocity changes corresponding to the assumed fundamental energy loss and its multiples. Coulomb interactions between the particles are neglected.

Figure 3(b) shows the simulation results, plotted as the effective electron energy loss spectrum as a function of the stretcher strength. We observe distinct spectral features to become visible above a stretcher field strength of $∼10^{7}$ V/m. Below that value, only the envelope of the spectrum is visible without the details. The higher the stretcher strength, the finer the resolution, but the total number of useful electrons in the spectrum decreases. Figure 3(c) shows cuts through Fig. 3(b) at four selected THz field strengths. We see an improvement of resolution as well as a decrease in signal.

The increase in resolution is a consequence of the sheared phase space shape created by the stretcher [see Fig. 3(a)]. The decrease in electron count is caused by the finite cycle duration of the compressing THz pulse. The stronger the stretching, the longer the electron pulses become at the compressor. If they are longer than one THz half-cycle, some parts of the electron pulse are not compressed and arrive continuously at the temporal focus [see Fig. 1(a)]. Hence, there is in the final EELS data a constant background in energy [see Fig. 3(b) at highest field strength]. Nevertheless, depending on the required measuring times for achieving a sufficient signal-to-noise ratio, down to 10 meV resolution can be realized for the given parameters.

Figure 3(d) summarizes these results. The energy resolution is taken from the simulations as the width of the zero-loss peak. We define the useful EELS signal as the number of electrons within the full-width half-max of the zero-loss peak and compare it to the case of perfectly linear phase space transformations for which there is no loss in signal. We see that the useful signal (blue) decreases in a nonlinear way with increasing stretcher field strength. The energy resolution (orange, FWHM) increases accordingly. A simple adjustment of the THz field strength at the stretcher can therefore be used to achieve the best compromise between signal strength and resolution for a given sample system.

Interestingly, the time resolution ΔT of a potential pump-probe experiment does not significantly depend on such an alignment. The reason is that the single-cycle pulses used for compression act as a temporal filter that only compresses such electrons that arrive within the linear slope of the field around the zero crossing of the central half cycle. All other electrons only produce a constant background with neither energy nor time resolution. Figure 3(e) depicts the effective time resolution that can be expected for the chosen parameters. We see that for negligible stretching (<10^5^ V/m), the time resolution remains 100 fs, the initial electron pulse duration after the gun. For stronger stretching, ΔT becomes worse as the energy resolution improves [compare Fig. 3(d), orange line]. Interestingly, if the stretching is further increased, the time resolution again improves, now mainly at cost of the reduction of signal strength [compare Fig. 3(d), blue line]. This effect is different as compared to microwave approaches^30^ and a direct consequence of the pulsed, single-cycle nature of our THz fields. Summarizing, it means that in our case, adjusting the energy resolution far beyond the cathode bandwidth limit always comes at the cost of signal strength. However, in situations where low signal is tolerable, the approach allows to have extraordinary high resolution in both energy and time, fundamentally limited only by the uncertainty principle.

## CONCLUSIONS AND OUTLOOK

In conclusion, we have shown an all-optical approach to measure energy spectra of femtosecond electron pulses via THz-based time-of-flight measurement in a temporal focus. The inherent synchronization of THz pulses with a pump laser pulse renders the scheme as a useful tool for time-resolved EELS measurements on femtosecond and potentially attosecond time scales. In contrast to magnetic chicanes or related approaches, our concept is largely independent of the electron beam's transverse emittance. It can therefore well be combined with high-brightness, flat emitter sources^2^ which are useful for many pump-probe investigations of complex materials. In dense multi-electron pulses, linear stretching can be generated via the space charge expansion of elliptical electron packet shapes^46^ instead of the THz-stretcher used here.

In practice, the presented concept offers a compact, simple, high-resolution pump-probe electron energy loss or gain spectrometer with table-top dimensions and without need for microwave electronics. One can conceive upcoming applications in concurrent research on, for example, time resolved electron spectroscopy,^20,47^ quantum metrology of electron pulses,^24^ attosecond pulse generation,^13–15^ or photon induced near-field microscopy.^22^ In waveform electron microscopy,^48^ where electrical field vectors are detected in space and time, an additional energy resolution such as achievable with the presented approach could reveal, in addition, the longitudinal field components in a direct way.
